# Driving the determinants of older people’s mental health in the context of urban resilience: a scoping review

**DOI:** 10.1186/s12877-023-04387-y

**Published:** 2023-11-02

**Authors:** Sajad Vahabi, Azadeh Lak, Niloofar Panahi

**Affiliations:** 1 Department of Urban Planning, Faculty of Arts, Tarbiat Modares University, Tehran, Iran; 2 Department of Urban Planning, Faculty of Arts, Shahid Beheshti University, Tehran, Iran

**Keywords:** Older adults, Health, Mental health, Scoping review

## Abstract

**Background:**

Population aging is a pervasive phenomenon occurring rapidly worldwide, while sustainable development goals are considered the mental health among older adults.

**Methods:**

To investigate the factors affecting mental health, we conducted a scoping review of the 47 papers published between 2015 and 2022 to explore various dimensions affecting older adults’ mental health.

**Results:**

Our finding mirrors four dimensions of creating healthy and sustainable environments for older adults: person, place, processes, and resilience-related health in the living environment. The person dimension includes individual characteristics, attitudes and behaviors, and health status. The place dimension is divided into five categories: land use, access, physical form, public open spaces, and housing, while the process consists of the social, cultural, and economic environments. Resilience-related health dimension emphasizes the impact of natural and man-made disasters on older people’s mental health.

**Discussion:**

These findings can provide policymakers insights into developing community-based environmental intervention strategies to promote mental health among older adults and support healthy and active aging.

**Supplementary Information:**

The online version contains supplementary material available at 10.1186/s12877-023-04387-y.

## Background

There are more than 703 million older adults over age 65 worldwide. The increasing population of older people is caused by a decrease in the fertility rate and increasing life expectancy [1]. East and South-east Asia have the world’s largest aging adult population, with more than 260 million people, followed by Europe and North America, with more than 200 million people. By 2050, the older population will double to 1.5 billion [2]. Moreover, urban residents, that is, the people living in urban areas, comprise more than 55 percent of the world's population. Statistics predict that by 2050, more than 68% of the world's population will live in cities [3].

Moreover, the Organization for Economic Cooperation and Development (OECD) shows that the share of the population of people over 65 years old will increase to 25.1% in its member countries in 2050. Thus, cities particularly have many older residents, about 43.2% of the older adult population [4], indicating the coincidence of two significant phenomena of population aging and urbanization in the coming years. The Sustainable Development Goals (SDGs), developed by the United Nations, emphasize the health and well-being of all, especially older people [5]. The World Health Organization defines healthy aging as creating and sustaining functional capacity that leads to well-being in old age [6].

Recently, the mental health of older adults has been considered one of the significant issues that attracted the attention of researchers and policymakers. The World Health Organization (WHO) defines mental health as a state of well-being in which each person can realize their potential, cope with the stresses of life, work productively, and contribute to their community [7]. Hoisington et al. (2019) mentioned that Mental health includes emotional and social well-being components as well as social skills and cognitive functions affecting participation in basic tasks and social roles [8]. According to the Global Burden of Disease Survey (2015), depression and anxiety are the leading mental illnesses (ranked third and ninth, respectively) that cause the most problems worldwide, associated with various health problems, such as cardiovascular diseases and reduced quality of daily life, particularly among older people [9]. Older people’s mental health is becoming a growing public health concern [10].

Although national patterns of depression have been documented, research about older people has received less attention [11]. Specifically, neurological and psychiatric disorders account for 6.6% of all disabilities, the fifth leading cause of disease in the older population [12].

According to [13], with increased social participation among older adults, more time in physical activity, and more frequent contact with neighbors and children, their depression can be reduced [13]. Ko et al. 2019 believed that older men were significantly more likely to report feelings of loneliness, depression, and frequent suicidal thoughts than women. Older women were more likely to report higher stress levels and depressive symptoms. Older people who work suffer from more stress and, at the same time, experience fewer depressive symptoms [14].

Furthermore, the built environment is potentially involved in the mental disorders of older adults through its effects on social communication, access to green space, exposure to noise, traffic, or air pollution, and changes in individual behaviors, such as physical activity [15]. Evidence shows that the physical and social dimensions of the neighborhood environment play an essential role in older peoples’ health to predict health outcomes beyond individual deprivation and psychosocial characteristics [16]. For instance, according to research, older people living in pedestrian-friendly urban areas (with easier access to facilities, beautiful scenery, open spaces, and road safety) are generally more physically active and healthier [17]. Furthermore, older adults are more vulnerable to stressors or risks due to reduced physical performance against environmental barriers [18]. Compared to younger adults, older adults appear to be more vulnerable to changes in the built environment [19].

Today, urban resilience is considered a paradigm affecting all aspects of human life. Meerow (2016) proposed a new definition for urban resilience be defined as the following: “Urban resilience refers to the ability of an urban system and all its constituent socio-ecological and socio-technical networks across temporal and spatial scales to maintain or rapidly return to desired functions in the face of a disturbance, to adapt to change, and to quickly transform systems that limit current or future adaptive capacity [20]”. They added socio-economic dynamics such as public health, monetary capital, demographics, and Justice and equity to shape the other subsystems. The livelihoods and capacities of urban citizens have a significant role in enhancing urban resilience. Furthermore, after the COVID-19 outbreak, urban resilience is critical to urban and human health. Resilience is the capacity of a system or community at risk to withstand, adapt to, and recover from the effects of a hazard in a timely and effective manner [21].

The COVID-19 outbreak caused the disconnection of older adults from the outdoor environment, and older people with a history of depression reported higher levels of depression or sadness during the pandemic [22]. Fear of being infected with the virus, hospitalization of family members, disconnection of the senior from social support, and fear of losing a job can also affect older people’s mental health [22–24]. Although some studies show that younger people were more emotionally involved than seniors in disasters such as the COVID-19 pandemic, others show that older people suffer a more significant threat and death from catastrophes. Additionally, some authors have discovered a relationship between emotional well-being, resilience, and social support among caregivers exposed to social isolation [25].

In recent years, many studies have measured the impact of social, economic, health, individual, and demographic factors on the mental health of older adults; however, there is still no comprehensive model for measuring the factors affecting older adults' mental health in urban areas. This review study aims to find the factors affecting the mental health of older people living in urban environments by reviewing papers published in the last seven years by focusing on understanding the general determinants of older people’s mental health in the cities as a narrative systematic review. This study provides a deep understanding of the factors affecting the mental health of older adults for policymakers to make plans to increase the mental resilience of older adults and improve their quality of life.

## Methods

A scoping review is a good tool for identifying domains or covering a body of literature on a particular topic, identifying knowledge gaps, existing literature, and concepts [26]. Arksey and O’Malley used a systematic scoping review for the first time in five main steps: identifying research questions, identifying related studies, selecting studies, charting and collecting data, and summarizing and reporting research results [27]. This method was then improved by Daudt et al. 2013, Levac et al. 2010, and Colquhoun et al. 2014 [28–30]. This study followed the PRISMA extension for scoping reviews (PRISMA-SCR) [31]. In recent years, some studies have been conducted on older people's mental health, each focusing on specific dimensions. Due to the complex nature of mental health, the scoping review is used in this study to identify knowledge gaps and reveal various factors affecting older people’s mental health.

### Identifying research questions

The first step in the scoping review is to identify research questions to reveal and link them to the research objectives. This study aims to review the papers on the mental health of older adults to identify the factors that can affect the mental health of older adults living in the urban environment in the face of stressful aspects (conditions such as COVID-19) and lead to an increase in urban resilience among older people. For this purpose, the following questions have been used to refine the research.

1. What factors affect Older adults’ mental health living in urban environments?
2. What factors affect Older adults’ mental health living in the city In facing disasters and changes, ‘for instance, COVID-19’, to achieve urban resilience?

### Identify related studies

We searched Scopus, PubMed, Web of Science, and Google Scholar for relevant papers from 2015 to 10 May 2022. Search strategies drafted by a professional person and refined through team discussion. The search terms were “mental health” and / OR “neighborhood” and / OR “urban area”, and “environment” and / OR “older people” and / OR “social environment” and / OR “built environment” and / OR “depression” and “anxiety” and “stress” and “Covid -19”. Article in Press in Scopus Database, Rural context-based, and non-English papers were excluded.

### Selection of studies

One thousand nine hundred ninety-four papers were collected after searching the databases. First, 280 duplicate papers were removed, and then, based on the analysis of abstracts and titles, 1646 other papers were removed from the study. After a complete review of the papers, 21 were removed from the list due to their relevance to the rural environment, lack of suitable sample sizes, failure to calculate mental health or mental well-being, and relevance to nursing homes. To increase consistency among reviewers, All reviewers screened the first 60 publications and discussed the review’s screening and result.

**Fig. 1:**
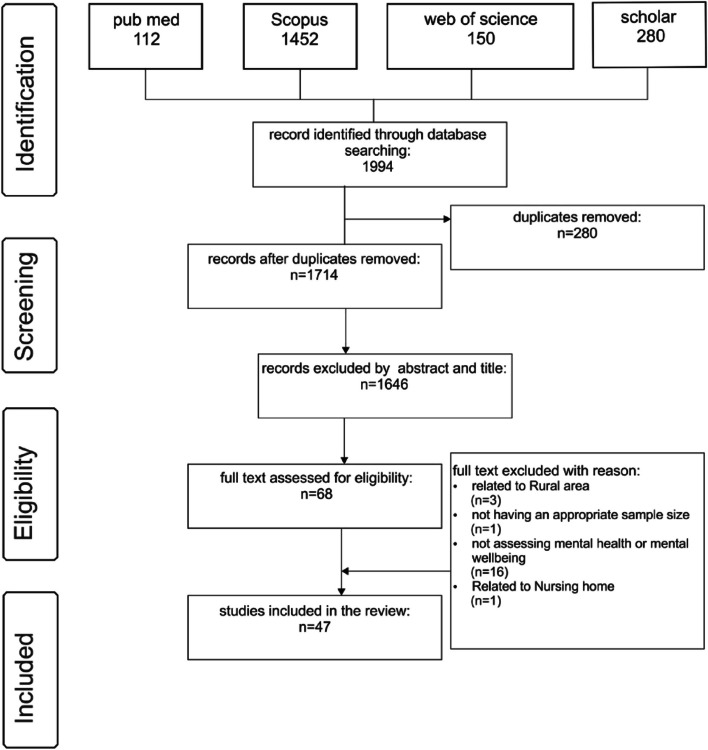
PRISMA flowchart for study selection

Three authors did the initial review of the abstracts and titles, and after reviewing, all authors decided which paper would remain for further study. After this process, 47 papers complied with the criteria. It should be mentioned that 4 of the documents were review papers. The article selection process, the PRISMA process, is presented in Fig. 1.

### Charting and interpreting data

For data extraction and charting, this study follows [28, 30] recommendations for scoping review data extraction and charting. In this regard, the authors chose the narrative review approach for data charting, as described in [30]. Based on this, Two reviewers developed an Excel file to examine which data needs to be extracted. Then, all related data were collected in an Excel file after selecting the papers. The Data included authors, year, geographical location, research scale, method, mental health measurement tool, personal factors, health factors, physical environment factors, social environment factors, economic factors, age of participants, and key findings.

#### Collecting, summarizing, and reporting the results

The last stage of the scoping review is collecting, summarizing, and reporting the results. Thematic analysis was used to combine, categorize, and codify the studies’ findings regarding the research questions and aims. Thematic analysis of the papers was done with an emphasis on the factors affecting older people’s mental health, such as the dimensions of the built environment, social environment, individual characteristics, and health status. Table 1 presents the categorizing process used in data analysis and coding.

**Table 1:** Themes of older adults mental health extracted from the scoping review

Dimensions	Sub-Dimensions		Indicators	Definition	References
**Person**	**Personal characteristics/determinants**		**Age**		[9, 13–16, 22, 33–37, 40, 46, 48, 50, 51, 53, 61–66]
			**Gender**		[9, 13, 14, 16, 22, 33, 35–37, 43, 46, 51, 61, 64–68]
			**Education level**		[14, 34, 36, 48, 51, 61, 62, 64, 66–68]
			**Ethnicity**		[9, 16, 34, 61, 64, 66]
			**Marital status**		[9, 13, 16, 32, 34, 37, 44, 48, 61, 62, 64–66]
			**Occupation**		[16, 33, 36, 65]
			**Household size**		[13]
			**Sedentary lifestyle**		[37]
			**Mutual-support**		[69]
			**Living arrangement**		[35]
	**Behavioral attitude/ determinants**		**Cigarette smoking**		[14, 61] [13, 14, 68]
			**Alcohol consumption**		[13, 14, 36]
			**Practicing exercises/ kind/frequency/length of activity**		[68]
	**Health Status**		**Physical health status**		[13, 22, 36, 39, 50, 64]
			**Pain feeling**		[68]
			**Well being**		[16]
			**Functional ability**		[64, 65]
			**Self-reported health**		[9, 44, 66]
			**Body mass index/obesity**		[34, 37, 39]
			**Activities of daily living limitation**		[61]
			**Chronic diseases**		[9, 61, 65]
			**Comorbidity**		[14]
			**Cardio-respiratory health**		[53]
			**Depression history**		[66]
			**Cognitive function**		[65]
			**Taking Antidepressant medication**		[10]
**Place**	**Subjective Attributes**	**Land_use**	**Service proximity (buffer)**		[34, 43]
			**Public facilities**	Community facilities, cultural facilities	[50, 52, 65]
			**Land use mix diversity / land-use composition**	Amenities and facilities, such as the library, community center, and local shops, traditional clinics, community outreach projects	[9, 35, 37, 39, 52, 65, 69]
			**Exercise, sports, and recreation facilities**		[35, 61]
		**Access**	**Connectivity**	Directness and availability to different areas in a region, composed of the street system, sidewalk network, pedestrian volumes, and directness of route	[35, 39]
			**Accessibility services**	The proximity of the home block and its neighborhood amenities Systems that provide connections between activities	[35, 40, 42, 43, 65, 69]
			**Physical activity/ walkable environment/**	Pavements and roads; safe pedestrian crossings Pedestrian infrastructure, good sidewalks, the surface area of open space,	[38, 42, 43, 51, 64]
			**Mobility**	Exterior and interior accessibility Ease of activities, convenience, disabled facilities, and comfortable movement	[48]
			**Transportation (public)**	Adequate and affordable public transport; bus stops	[52, 53, 65]
		**Physical form**	**Population density**	free from crowds	[9, 36, 43]
			**Safety in the built environment**		[39, 44, 50, 70]
			**Access to nature and green spaces**	Contact with nature, green spaces, parks, gardens, micro-climate	[45]
			**Physical barriers**	freeways, railway lines, rivers, canyons, hillsides	[18]
			**Topography/slope**		[65]
			**Proximity to roads**		[15]
			**physical permeability**		[39]
			**Familiarity with environment**		[33]
			**Perceived aesthetic/environmental attractiveness**	Attractiveness and appeal of a place	[39, 42, 67]
			**Natural landscape**		[67]
			**Intersection density**		[43]
			**Neighborhood Safety**		[38, 42, 48, 67]
			**Residential density/density of housing**		[35, 41]
		**Public open spaces**	**Street lighting**	Outdoor Lighting	[37, 53]
			**Security to crime**		[10, 18, 41, 67]
			**Social disorder**	not belong, not trust, unfriendly, and no help	[66]
			**Availability of blue space**		[10]
			**Recreation/ public open spaces**		[35]
			**Cleanness/lack of littering/vandalism/decay**	Cleanliness, visual attractiveness,	[39, 70]
			**Pollution (air, visual, noise, litter…)**	fresh air, free from noise and congestion	[39, 41, 67, 70]
			**Proportion of overcrowded**		[10]
			**Landscape**	Outdoor seating/urban furniture/ Seating area for rest	[37, 39]
			**outdoor quality**		[49]
			**Coverage of blue and green spaces**		[10]
			**Restoration Serene/ Calm/peace**	the environment is silent and calm; No contact with many people; Not disturbed by traffic noise	[46]
			**Restoration Nature**	An environment with natural qualities; Wild and untouched; Free growing	[46]
			**Restoration Social**	Vistas over the surroundings; An abundance of people and movements in the environment; Possible to watch entertainment or exhibitions	[46]
			**Prospect**	An environment with open views; Vistas over the surroundings; Plane and well-cut grassy surfaces	[46]
			**Restoration Refuge**	An environment with many bushes; One can sit and watch other people being active	[46]
			**Feelings of renewal**		[45]
		**Housing**	**Housing quality variable**		[50]
			**Neighborhood Safety**		[38, 42, 48, 67]
			**Residential density/density of housing**		[35, 41]
			**Household facilities**		[49]
			**Type of housing**		[49, 61]
			**The length of residence**		[16]
			**Indoor space layout**		[49]
			**History of house**		[49]
**Process**	**Social environment**		**Quality of life/well-being**		[16, 62]
			**Social safety**		[70]
			**Proportion female**		[10]
			**The ratio of older people population in the neighborhood**		[42, 61, 70]
			**Social interaction**	Community and social participation/interaction/relation, sense of community, community building, and sense of belonging	[13, 33, 37, 48, 65]
			**Feeling lonely**		[37, 48]
			**Social housing**		[71]
			**Social support/ community life facilities and services**		[38, 51, 66, 69]
			**Education, learning, employment, and volunteering,**		[35]
			**Social network types**	diverse social network, family social network restricted social network	[32]
			**Social capital “Bonding capital.”**		[40]
			**Social capital “Bridging capital.”**		[40]
			**Quality of social ties**	the number of relatives and friends who can offer help when respondents are in need	[64]
			**Quantity of social ties**	the number of relatives and friends they can meet or contact at least once a month	[64]
			**Social trust/ Social cohesion/**		[38, 42, 48, 63, 72]
			**Social engagement**	1-interactions with neighbors, two participate in volunteer work	[72]
			**Social contact**	meeting relatives; meeting friends; talking to neighbors	[53]
	**Cultural Environment**		**Social activity**	Forms of recreation, such as walking and other exercises	[50] [52]
			**The sense of place: place attachment/ place identity**	Heritage, sense of place, the importance of local identity, and cultural components integrated into the planning and management of the site	[51]
	**Economic Environment**		**Health care services**		[54]
			**Sncome/pension**		[10, 69]
			**Socio-economic status**		[16, 35, 40–42, 44, 61]
			**Car ownership**		[53]
			**Homeownership**		[61, 67]
			**Household income**		[13, 36, 47, 50, 51, 65]
**Resilience-related Health in the Environment**	**Infectious disease covid-19**		**Personally affected by own or other’s illness, deaths**		[55]
			**Feelings of being fearful, hopelessness, powerlessness, annoyed, frustrated**		[24]
			**Worried about having the disease**		[23]
			**The decline in Activity engagement**		[56]
			**Worried about the pandemic**		[55]
			**Support needed but not received**		[55]
			**Contact frequency with children/Others**		[55]
			**Being hospitalized**		[22]
			**Physical disconnection**		[24]
			**Disrupted all types of social support**		[24]
	**Disasters**		**Worrying about storms**		[57]
			**loss of friends and family members**		[58]

## Results

Table 2 shows the characteristics of the reviewed papers. Twenty-seven studies (57.4%) are from Asia, ten studies (21.3%) are from Europe, and six studies (12.8%) are from America. In addition, the most significant number of studies is related to the Chinese mainland, with 11 (21.4%) studies, and Hong Kong, China, with eight studies (17%). Also, 41 (87.23 percent) studies were conducted with the quantitative method and 2 (4.25) with the qualitative approach. Four studies are review articles.

**Table 2:** Characteristics of the reviewed studies

Study characteristics	No	%
Publications year (2015–2022)		
2015–2017	6	12.8
2018–2019	9	19.1
2020–2021	26	55.3
2022	6	12.8
Study design		
Qualitative study	2	4.25
Quantitative study	41	87/23
Review papers	4	8.52
Study country and region		
Asia (continents)	27	57.4
Chinese mainland	11	23.4
Hong Kong, China (region)	8	17
Other countries	8	17
America (continents)	6	12.8
US	4	8.5
Canada	2	4.3
Europe (continents)	10	21.3
Uk	4	8.5
Other countries	6	12.8
Without case study	4	8.5
Study participants age		
50 and more	4	11.8
60 and more	14	41.2
65 and more	11	32.3
Other age range	5	14.7
Categories		
Personal	32	68.08
Place	35	74.5
Process	35	74.5
Health, Resilience-based Environment	5	10.6

Analysis of assessment tools of mental health consisting of CES-D (*n* = 11) and GDS-15 (*n* = 9) was used in these studies to measure older adults' mental health. Tools such as HADS, UCLA, MSC, SF-12, SF-36, PSS, MMSE-2SV, SWLS, and GAI were also employed. Table 3 shows the mental health measurement tools used in the papers.

**Table 3:** Mental health measurement tools used in the papers

Study measures	No	%
CES-D	11	23/405
GDS-15	9	19/15
UCLA	4	8/51
PSS	2	4/255
SF-12	2	4/255
SF-36	2	4/255
HADS-A	2	4/255
GAI	2	4/255
SWLS	2	4/255
MMSE_2SV	2	4/255
Others	9	19/15

**Fig. 2:**
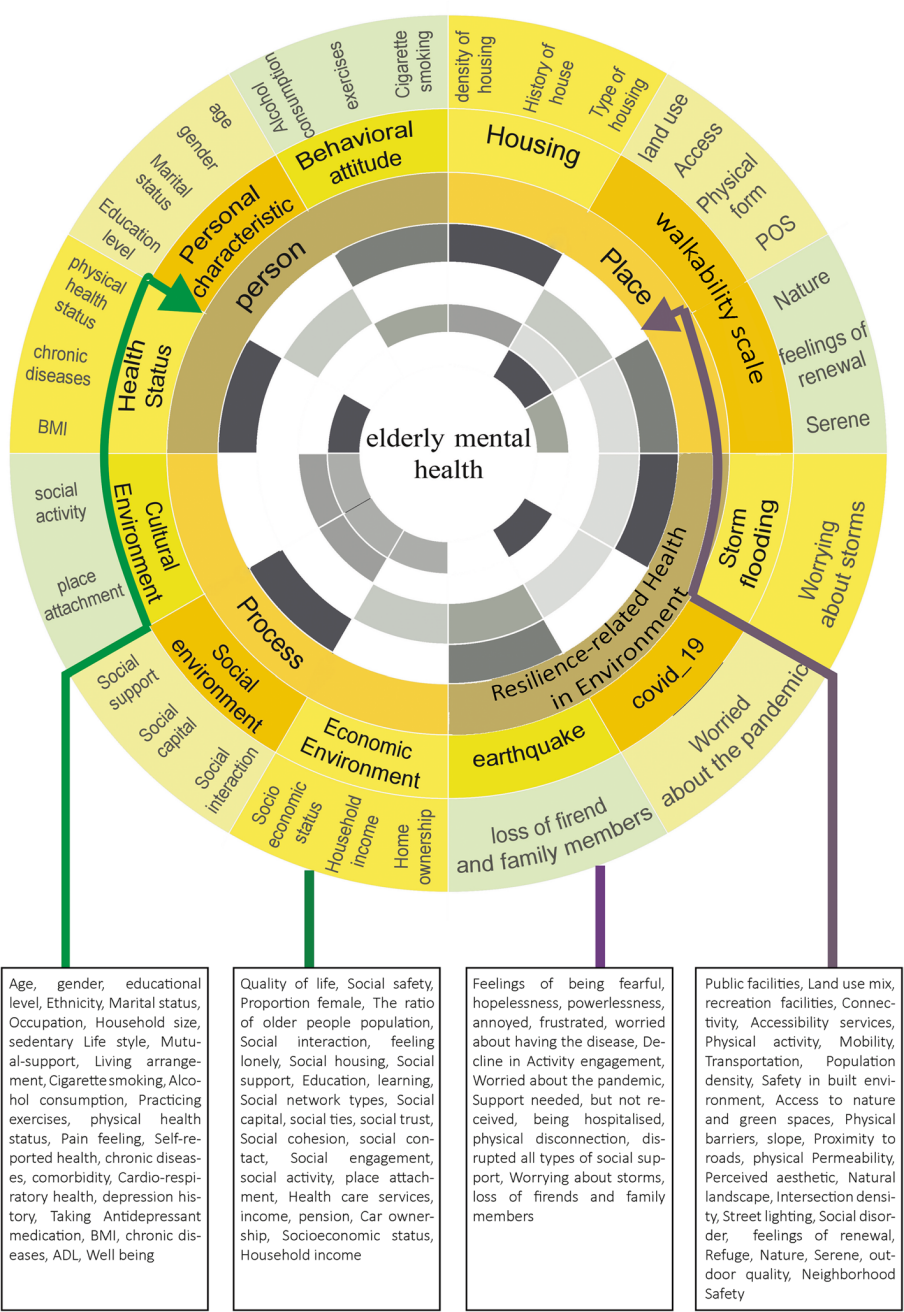
Determinant Model of older people’s mental health

Older people studied in the papers were generally aged 60 and over (41.2 percent) and 65 and over (32.3 percent). Additionally, more than 74% of the studies mirror the environment dimension and its impact on mental health, as 46% examined the effects of the social environment on the mental health of older adults. In 14 papers, the impact of economic factors on older people’s mental health was studied; however, 46% of the papers considered personal health and its role in mental health. 46% of studies examined personal characteristics and their effect on mental health. Ten studies considered factors such as physical activity, social activity, the proportion of older people population, social relationships, household income, and social capital as mediating factors affecting older people’s mental health. Three papers examined the cultural aspects and their impact on mental health, and five studied the social health of older people against disasters, especially the COVID-19 virus. The main dimensions and codes are summarized in Fig. 2.

**Fig. 3:**
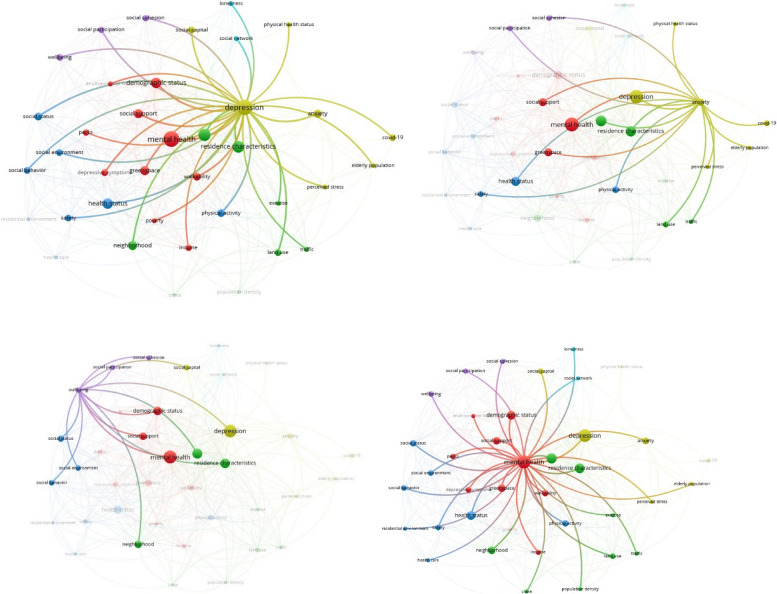
Term co-occurrence map for different mental health disorders from top-left: depression, anxiety, well-being, and overall mental health

According to the findings, intricate interactions between factors were investigated through the Co-occurrence of keywords with VOS Viewer for four mental health disorders reviewed in this study: mental health, well-being, anxiety, and depression. Each of these shows the different relationship between mental health and environmental determinants. Mental health factors include social support, social cohesion, social network, social capital, social status, land use, traffic, Healthcare, crime, income, health status, social environment, social behavior, residence characteristics, exercise, population density, green space and parks, residential environment, safety, neighborhood, perceived stress, demographic status, older adults population. Figure 3 shows the network visualization map.

Well-being is linked to social participation, cohesion, social support, social capital, demographic status, residence characteristics, social environment, and social behavior. Depression disorder is related to physical health status, walkability, health status, safety, income, neighborhood, physical activity, safety, land use, traffic, residence characteristics, green space and parks, social participation, social cohesion, social support, social capital, social network, demographic status, loneliness, poverty, neighborhood, social status. Anxiety disorder is connected to social support, social participation, social cohesion, residence characteristics, physical activity, older adult population, health status, land use, traffic, green space, COVID-19 outbreak, perceived stress, and safety. Additionally, as shown in the Co-occurrence term, depression highly impacts older people’s mental health.

**Fig. 4:**
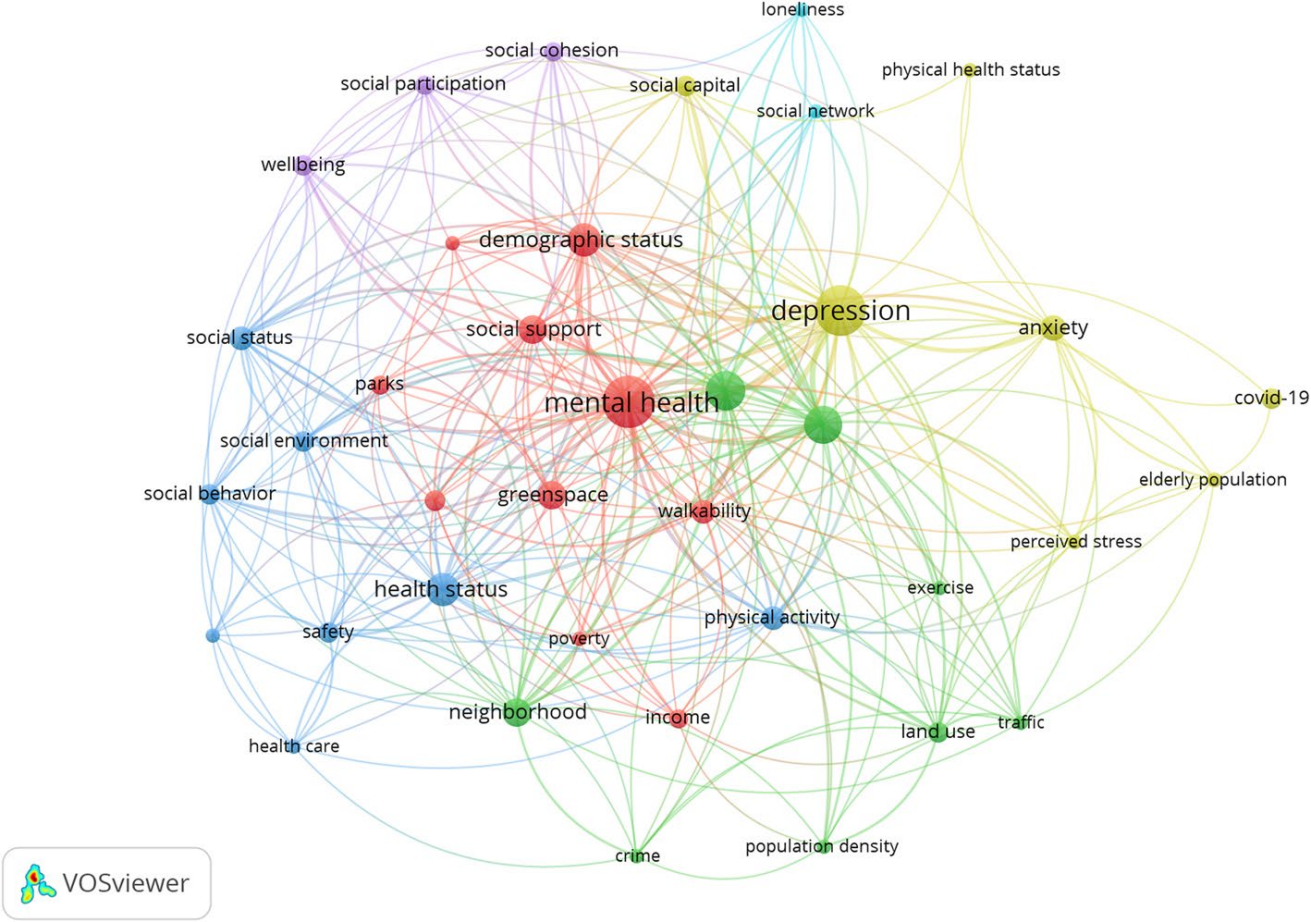
Term co-occurrence map of mental health disorders

The above findings explain that the various dimensions of mental health in older people are most influenced by environmental, individual, social, personal health, and economic factors. The paper’s review showed that older people’s mental health was associated with many factors, including the built and social environment. After thoroughly reviewing the papers, the factors affecting older people’s mental health were identified, and concepts related to various environmental dimensions were coded. The dimensions and criteria based on the Vos viewer are presented in Fig. 4.

### Personal dimension

This dimension includes three sub-dimensions: personal characteristics, attitudes and behaviors, and health status. The following criteria and definitions of individual determinants have been extracted and coded from 32 papers. The papers have considered personal factors such as age, gender, education level, ethnicity, race, house ownership, employment, marriage, household size, sedentary lifestyle, mutual support, and living arrangement [32–35]. Three studies reported that living in areas with a high percentage of older people directly impacted older people’s health. Domènech-Abella et al. (2020) stated that a sedentary lifestyle and less physical activity reduce older people’s mental health. Attitudes and behaviors comprise three components: smoking, alcohol consumption, and physical exercise [13, 14, 36]. Byeon (2019) showed that older people who do flexibility exercises for an average of 1 to 4 days are 81 percent less likely to develop depression than those who do not. People who smoke are more likely to experience high-stress levels and depression [14].

Lam et al. (2020) showed a close relationship between personal characteristics such as physical and body weight and mental health. Moreover, individual health factors include physical health, well-being, pain sensation, functional ability, self-assessment of health, body mass index (BMI), restrictions on daily activities, chronic diseases, co-occurrence of multiple disorders, and lung diseases [31, 35, 39, 47]. Therefore, people with a good BMI will be healthier and experience better mental health. Also, having two or more diseases simultaneously affects mental health [14].

### Place-based dimension

The place dimension can affect older people’s mental health to a large extent, which is presented in 35 studies. The place-based features include land use, access, physical form, public open spaces, and housing. Eleven studies focused on land use, including proximity to services, public facilities, land use mix, and sports facilities [34, 37]. Koohsari et al. (2019) report that women with better access to transportation stations have a higher level of mental health. Commercial and recreational facilities directly impact the mental health of older adults. The access factor is considered in 13 studies and includes connectivity, service access, mobility, pedestrian environment, and public transportation [38–40]. Low walkability of the built environment increases the chances of loneliness, which is associated with mental health [37].

Additionally, physical form, which was considered in 14 studies, includes population density, safety, access to green space, physical barriers, slope and topography, proximity to roads, physical permeability, familiarity with the environment, Environmental aesthetics, natural landscapes, and residential density [41–43]. Perception of safety is related to the physical and mental health of older people [44]. Furthermore, decreasing physical barriers and crime in the neighborhood increase older people’s mental health [18]. Public open space was considered in 13 papers, paying attention to issues such as street lighting, safety against crime, social disorders, availability of water spaces, recreational spaces, environmental cleanliness, noise pollution, congestion, Landscapes, outdoor quality, green and blue space coverage, restoration Serene, nature restoration, social restoration, landscape, restoration refuge and a sense of renewal [41, 45, 46].

More importantly, public open spaces significantly impact older people’s mental health, so green and blue infrastructures in the neighborhood and near beaches and lakes are associated with reduced use of antidepressants [10]. Green spaces are also associated with increased physical activity, improving mental health perception and well-being, and promoting older people’s mental health [34, 47]. Ten papers consider housing quality, neighborhood safety, housing density, housing facilities, type of housing, length of stay, interior design, and house history [38, 48, 49]. The quality of housing and the neighborhood’s safety affect older adults' mental, physical, and mental health [50]. External building characteristics, interior design, home facilities, and interior home space profoundly impact the mental health of older people [49].

### Procedural determinants

Procedural dimensions include social, cultural, and economic environments. The social environment affects the mental health of older adults in various ways. Chen et al. (2016) stated that older people, with the support of friends or family, have better mental health; additionally, social capital can play a supportive role in mental health [27]. The essential components of the social environment are presented in 21 papers, including quality of life and health, social safety, the ratio of women in the neighborhood, the ratio of older people population in the neighborhood, social interactions, feeling lonely, social housing, social support, education, learning, social network type, different types of social capital, Social nodes, trust, and social cohesion [34, 38, 51, 55].

Besides, the cultural environment was repeated in three papers. Older people’s participation in social activities reduces stress and depression in older people [14]. This sub-theme profoundly affects the mental health of older adults and includes two criteria for social activities: the sense of place and identity [51, 52].

According to our review, social and economic status is the most critical component of the economic factor. The economic environment is studied in 15 papers related to health: income/retirement, socio-economic status, car ownership, housing ownership, and household income [42, 53, 54].

### Resilience-related health in environment dimension

Natural disasters and crises profoundly affect mental health. Five papers specifically address the issue of Covid-19 and its impact on older adults’ social health and resilience. With the Coronavirus outbreak, many older people were forced to stay home. Lockdowns and cocoons caused some problems profoundly affecting older people’s mental health. Dimensions introduced in these studies include cases of being affected by COVID-19, feeling lonely, fear, despair, helplessness and fatigue, fear of getting COVID-19, decreased participation in activities, need to receive support, frequent communication with others, hospitalization, disconnection, and loss of social support [23, 24, 55]. Older people’s physical activity has significantly decreased during the pandemic; thus, the severity of depression in women has increased [56]. Two studies have examined the impact of disasters on mental health, which refer to the fear of storms and the loss of families, respectively [57, 58].

## Discussion

This study has used a scoping review to identify, select, and combine the findings of studies that have examined determinants affecting the mental health of older adults in urban areas in the context of urban resilience. We found that urban resilience refers to a city’s capacity to support its citizens and systems. Like humans, cities are resilient due to a variety of intricate variables. Surprisingly, however, there is a conceptual link between urban and psychological resilience because both ultimately benefit the person or citizen. As a result, the elements that contribute to urban resilience may also contribute to mental resilience and vice versa.

As a result, the conceptual similarity between urban and psychological resilience is rational, and the distinct characteristics that distinguish the two are likewise connected. Researching these elements and looking at resilience’s impact on mental health in urban settings would be fascinating. Based on reviewing the previous paper, we introduce the ideas of older adults’ mental health and urban resilience, describe urban resilience's functional link with older adults’ mental health, and pinpoint the characteristics of urban resilience in four dimensions of determinants associated with mental health in older people (anxiety, depression, mental health, and well-being), including personal and place-based factors, processes in the living environment (social, cultural, and economic), and environmental health (natural and man-made disasters).

Urban Resilience focuses on the system’s ability to maintain environmental harmony despite perturbations. Additionally, it aids in the recovery of individuals from disturbance. It deals with the capacity to adjust to and respond to structural change throughout time. Thus, most studies have pointed out the effect of older people’s characteristics on their mental health as the most influential factor. Regarding socio-economic processes, studies have emphasized the role of social capital, social interactions, perception of social security, and the economic status of older people in improving their mental health of the older people. This study scrutinizes the mental health of older adults by analyzing the geographical scope, tools for measuring older people's mental health, and key findings on the main reported determinants of older people’s mental health to provide comprehensive knowledge about the consequences of the results and gaps which can be helpful for physicians, researchers, and aging policymakers.

Based on the role of urban structure and place in building urban resilience, the place and land use range mix has been found in most studies. Still, some individual variables that refer to walkability in these studies (such as crosswalks, barrier-free sidewalks, and recreational environments) have a significant relationship with health. In particular, the presence of green space/park was the most studied variable in all areas related to mental health outcomes. A few studies paid attention to individual and place-based determinants. In contrast, the qualitative studies paid more attention to the perceptual characteristics of the quality of housing, the perception of environmental security, and the perception of the social environment.

In addition, perceptions of environmental health, fear of infectious diseases such as COVID-19, and injuries caused by natural and man-made disasters were considered in the papers. Most studies that looked at the impact of individual and environmental factors on older people’s mental health found significant relationships, with evidence supporting the moderating effects of demographic characteristics, health status, health behaviors, neighborhood walkability, and an area’s level of development intensity. The mediating results of both personal and place-based dimensions were not confirmed. Due to significant differences in research methodology, measurement methods, sample sizes, and neighborhood definition, direct comparisons between studies are impossible.

In addition, the results of the analysis of the keywords in VOS viewer showed that the study of factors such as Demographic Status, Health status, Social support, Green space, Walkability, Neighborhood Characteristics, Physical activity, Social Support, Social environment in research related to mental health is of great importance and has been studied more than the rest of the factors. Also, some factors such as traffic safety, safety, exercise, loneliness, social network, and physical health status have been less discussed.

### Methodological limitations of review studies

A- Lack of standard tools in measuring the determinants of mental health: Contradictory findings on the role of the four dimensions on older people’s mental health may be partly due to the lack of clarity in the definition of standard mental health measurement tools for the four dimensions of individual, place, process, and environmental health. This problem is the most prevalent in place-based features, which mention various tools to study the effects of place-based features on older people’s mental health. Other studies have pointed to the lack of standard tools in investigating the factors affecting older people’s mental health [59]. Studies such as Ivey et al. (2015) point to using common mental health tools to link mental health and neighborhood measurement metrics using various data [44].

b- Lack of evaluation of specific Place-based features: Most Place-based features studied in the papers, such as public parks, access to public transportation, and land use composition, are among the common features necessary for healthy aging. Although an increasing number of studies have studied the separated criteria instead of integrated criteria to portray the role of individual and Place-based components, there is still a knowledge gap about place-based features that may affect the mental health of older adults. A small amount of literature outside the scope of this study, including unreviewed documents, suggests potential design solutions for adapting outdoor environments to mental health. They consider some design dimensions such as distinctive architectural features, ample street facilities (such as shaded chairs and lighting), a clear hierarchy of locations, comprehensive, simple, unobstructed sidewalks, and short and distinctive pedestrian crossings. These place-based features are attractive targets for interventions because they are generally more practical and sustainable than large-scale environmental or program-based interventions. Future studies need to identify valid and consistent ways to measure the role of specific design features and establish more robust paths between place-based characteristics and older people’s mental health. For example, green space is an essential Place-based feature playing a significant role in promoting the health of older people, particularly mental health [60]. However, our findings indicate that the total area/ratio of green space alone may not fully show a significant impact on health outcomes, and future research should consider the morphology and quality of green space for better assessment. There are no specific measuring tools for housing quality for older people, especially social housing. Studies conducted in this regard examined the needs and preferences of older adults and their effect on older people's mental health using qualitative methods.

### Assessing the mental health of the older people

Mental health in older people has several definitions. Studies considered mental health in older adults as cognitive health; consequently, the loss of mental health is considered dementia in older people [59]. Another range of studies related to mental health emphasized some emotions such as stress, depression, and anxiety [15, 34, 43, 56, 61, 62]. Despite different emotions in older people, there was no standard and comprehensive tool for mental health testing in older people. The most used tools were CES-D and GDS-15, which were used to study the rate of depression in older adults, and the others, the emotional disorders, in older adults received little attention. Especially when health is endangered in conditions such as pandemics or natural hazards, these instruments have little efficiency in measuring older people’s mental health, and most studies have turned to qualitative studies.

### Study of mediating effects

Five studies looked at mediators focused on P.A. or walking outcomes using subjective self-reports, and three used objective measures (accelerometer measure of P.A.). In some papers, the mediating effects of subjectively estimated P.A. or walking were considerable. As respondents may not recall their previous physical activity accurately, this assessment approach frequently results in recall bias, limiting the capacity to find reliable relationships with cognitive function. Future studies should use objective techniques to capture P.A. levels more accurately and prove its mediation function in mental health conditions. Furthermore, more research is needed to understand the underlying mechanism of the long-term link between greenness and mental health. Depression was also discovered to mediate the relationship between urbanization and personal/crime-related safety [41].

In recent years, cities in North Africa and the Middle East have been experiencing special conditions under the influence of climatic phenomena. Every year, many people die due to dust. The vulnerable groups suffering from these changes are older people, who have to stay home because of the effects of these factors on health. Prolonged stay at home and disconnection from the external environment have affected older people’s mental health, so it is suggested that future research consider the effects of dust and climate change on older people’s mental health.

### Strengths and limitations of the research

This research uses a scoping review method and a systematic search strategy based on research questions. In this study, a selection of scientific evidence and studies conducted on the mental health of older adults living in the city can be used to strengthen older people’s mental health in cities. This study also facilitates the formation of future research with a comprehensive view and by identifying different dimensions of mental health in older people. There are several limitations to the scoping review in this study. First, In this research, only four databases in English have been examined, which means that studies from other languages have not been included. Second, this study did not investigate factors such as dementia, schizophrenia, bipolar disorder, cognitive disorders, and suicide related to mental health disorders in older adults. Third, in this study, only research with the urban context is considered, and older people living in rural areas are out of the scope of this research. Fourth, different dimensions of the impact of the COVID-19 pandemic on the mental health of older people under environmental health are presented in this study. We suggest that in future research, the effect of the COVID-19 pandemic on the mental health of older adults living in cities be examined in more depth. Fifth, the significant studies included in this study are from developed countries in Asia, Europe, and America, so the findings of this study may be less applicable in developing countries. Sixth, Some natural disasters, such as floods and earthquakes, have not been investigated in this study. Still, since these factors affect the mental health of older people in resilient environments and there is a need to study in this field, some of the effects of these factors are shown in 5 4 to emphasize this issue. Only two factors were considered influential factors in mental health to highlight the importance of catastrophe on older people’s mental health. It is suggested that these factors’ role in older people’s mental health be examined in more detail in future research.

## Conclusion

This study has presented a comprehensive and valid review of the papers about determinants of mental health of older adults in the context of the urban resilience approach that has been of interest in recent years. In this study, urban resilience is defined as the ability of the urban environment and its residents to maintain continuity in the face of shocks and stresses, especially for vulnerable older adults, so that they can adapt positively to achieve a sustainable environment. The results of this study have been presented in the form of a conceptual model that refers to the cognitive health component of older people, including personal and spatial dimensions, socio-economic and cultural processes, and health-oriented environments. The personal dimension refers to older adults’ demographic characteristics, health behaviors, and health status. The spatial dimension relates to land use, access, urban form, urban space, and the quality of older people’s neighborhoods.

Procedural components examine socio-cultural processes, economic conditions, and the policy-making and governance environment (including urban and health management). The difference between this study’s results and cognitive health components is the resilience-related health component, which tries to increase the adaptability of older adults in the face of shocks and disasters, including natural disasters and shocks caused by future infectious diseases that have affected the urban older people community during COVID-19. These components can make it possible to realize urban resilience with resilient residents.

Future related studies in designing a healthy and resilient environment for older people in a situation where climate changes, fine dust, and various phenomena resulting from global warming affect cities can effectively promote older people’s health and ultimately improve older adults’ health. The findings of this review can also provide insights for policymakers and those involved in planning and designing the development of environmental intervention strategies at the community level to promote mental health and resilience among older people and support healthy and active aging.

### Supplementary Information

**Additional file 1: Table 1.** Reviewed article characteristics.

## Data Availability

After request, supporting data and data analysis materials are available from the corresponding author (AZ).
