# Supplementary material for: Driving the determinants of older people’s mental health in the context of urban resilience: a scoping review
Source: BMC Geriatr. 2023 Nov 2;23:711. doi: 10.1186/s12877-023-04387-y (PMC10623797; doi:10.1186/s12877-023-04387-y)
Supplement: Supplementary file 1 — Additional file 1: Table 1. Reviewed article characteristics. [file 12877_2023_4387_MOESM1_ESM.docx]

**- Additional file**

| Additional file 1: Table 1 reviewed article characteristics | | | | | | |
| --- | --- | --- | --- | --- | --- | --- |
| No | **Auter name & year of study** | **country** | **methed** | **Study sample** | **M.H measurement** | Key findings |
| 1 | J Domènech-Abella, J Mundó, M Leonardi, S Chatterji, B Tobiasz-Adamczyk, S Koskinen, JL Ayuso-Mateos, JM Haro and B Olaya [39] | Finland, Poland, and Spain | Quantitative | 10,799 | UCLA_3  CIDI 3.0 | Being female (gender)  age  Widowed or never married  a sedentary lifestyle  Feeling lonely  Obesity  having several chronic physical conditions  social interactions  B.E. usability including: Land use mix, diversity, adequate public transport stops, Access radius to public transport, urban furniture |
| 2 | N Kabisch, C Püffel, O Masztalerz, J Hemmerling and R Kraemer [38] | Germany | Quantitative | 33 | SF_12  POMS | Age  Gender  Occupation  Familiarity with parks “environment”  Frequency of visiting park  Green environment  Social interactions |
| 3 | Y Zhao and PK Chung [10] | Hong Kong | Quantitative | 360 | SF_36 | physical barriers  less crime |
| 4 | MJ Koohsari, GR McCormack, T Nakaya, A Shibata, K Ishii, A Yasunaga, T Hanibuchi and K Oka [43] | Japan | Quantitative | 349 | GDS-15 | Gender  Population density  Availability of destination  Intersection density  Distance to train stations |
| 5 | DRY Gan [49] | - | Review | - | - | walkability  esthetics  trash  access to services, and park  safety  cohesion  percentage of seniors  poverty |
| 6 | SY Sohn, WT Joo, WJ Kim, SJ Kim, Y Youm, HC Kim, YR Park and E Lee [45] | South Korea | Quantitative | 814 | - | marital status  number of children  number of close relatives  number of friends  In-degree centrality  Out-degree centrality  Closeness centrality  Betweenness centrality |
| 7 | M Hanslmaier, A Peter and B Kaiser [37] | Germany | Quantitative | 2203 | - | Age  Sex  Education in years  Health status  walkability  natural surveillance  Social status and resources  Social capital  social support  place attachment  Perceived disorder |
| 8 | H Ko, YH Park, B Cho, KC Lim, SJ Chang, YM Yi, EY Noh and SI Ryu [36] | South Korea | Quantitative | 1023 | UCLA  GDS  MMSE-2SV  EQ-5D | Age  Gender  Marital status  Surviving child  Education level  Living alone  Religion  Health status  Social activity  Quality of life  Economic status |
| 9 | Y Liu, M Dijst, J Faber, S Geertman and C Cui [35] | China | Quantitative | 1035 | - | age,  gender  Physical health,  Physical functioning (Mobility, self care, Pain and discomfort, vidion)  Physical conditions (Asthma, Chronic lung disease)  physical activities (e.g. sports, fitness and leisure activities)  Housing quality  cultural facilities  Leisure activitie  Safety  social activities  Household income |
| 10 | S Tang, HF Lee and J Feng [34] | China | Quantitative | 591 | Self-reported mental health | Age  Street network density  Accessibility to public transport,  Accessibility to parks  Open space qulity  Socio-economic status  Bonding social capital  Bridging social capital |
| 11 | HJ Lee and DK Lee [15] | South Korea | Quantitative | 11408 | - | sex,  age,  educational level,  Being a basic livelihood social security recipient  Household type  Comorbidity  Physical activity  Cigarette smoking  Alcohol consumption  Participation in social activity |
| 12 | J Finlay, T Franke, H McKay and J Sims-Gould [52] | Canada | Qualitative | 27 | - | Blue and green space quality  Contact with nature  Feelings of renewal and rejuvenation |
| 13 | Y Guo, Y Liu, S Lu, OF Chan, CHK Chui and TYS Lum [33] | Hong Kong | Quantitative | 1553 | MCS  SF-12  SOC | Age  Gender  Marital status  Educational attainment  Housing Type  Living arrangement  Year of residency  street connectivity  Park-based green space  vegetation-based green space  Land use mix  Recreational services  Residential density  Design  Diversity  Destination  Subjective financial Sufficiency  availability of health services |
| 14 | L Qiu, Q Chen and T Gao [32] | China | Quantitative | 300 | PRS | gender  Age  landscape preference |
| 15 | J Miao, X Wu and X Sun [56] | China | Quantitative | 2715 | HSCL-10 | the proportion of the population with at least senior high school  education,  the proportion of the population in high-status occupations (i.e., managers, administrators, and professionals), and  the proportion of the population holding a rural hukou  social engagement  social cohesion |
| 16 | H Khosravi and SO Tehrani [50] | Iran | Quantitative | 411 | SRQ-20 | walkability  Spatial comprehension  safety  Calm, peace  social cohesion  social trust  social support  spatial stimulation = Spatial inclusiveness |
| 17 | C Li and Y Zhou [54] | China | Quantitative | 9143 | CES-D | Outdoor building characteristics  Building structure  History of building (years), and  Type of building  Indoor space layout  Household facilities  Outdoor quality |
| 18 | CW McDougall, N Hanley, RS Quilliam, PJ Bartie, T Robertson, M Griffiths and DM Oliver [6] | Scotland | Quantitative | 2 million | - | Proportion female  Antidepressant medication  availability of freshwater and coastal blue space coverage freshwater coverage,  Distance to large lake  Distance to coast  Public green space coverage  Total green space coverage  Urbanicity  Proportion overcrowded  Crime rate  Proportion state pension  Proportion low income |
| 19 | J Won, C Lee, SN Forjuoh and MG Ory [51] | - | Review | - | - | The ratio of the elderly population  General Neighborhood safety include, neighborhood safety or safety pertaining to walking, safety fo walk  crime related safety include ((crime incidents)),” “surveillance,” and “crime watch signage  traffic -related safety included safety from traffic,” “traffic volume, heavy traffic, teraffic coliision, traffic-control/calming devices,” “traffic speed,” and “crossing aids.”  proxies for safety domain included vandalism, graffiti, litter and rubbish, odors, trash on the street, stray animals, lack of or poor conditions of street lights, loud noise, and abandoned buildings |
| 20 | R Wang, Z Feng, Y Liu and Y Lu [14] | China | Quantitative | 10105 | CESD | Gender,  Age,  Educational attainment,  Marital status,  Household size  Smoking ,  Use of alcohol  Physical health status  Functional ability  Living area quality(rural or urban neighbourhood)  Social participation  Annual household income |
| 21 | VC Pun, J Manjourides and HH Suh [9] | USA | Quantitative | 5266 | UCLA -9 HADS-A  CESD-11  PSS-4 | Age  Diabetes  Proximity to roads  Physically active  Urbanicity |
| 22 | H Byeon [42] | South Korea | Quantitative | 327 | PHQ-9 | Gender  Education  Smoking  Pain and discomfort  walkability index  Economic activity |
| 23 | VC Pun, J Manjourides and HH Suh [31] | USA | Quantitative | 4118 | PSS-4  HADS-A  CESD-11  UCLA-9 | Age  Gender  etnecity  Education  Season  Widowed, Divorced  (BMI)  Greenness |
| 24 | WWY Lam, BPY Loo and R Mahendran [47] | Hong Kong and Singapore | Quantitative | 347 | MMSE  GDS-15  SF-36 | Physical health  Body mass index “obesity”  Size of parks  Land use mix  Comfort: (population density, quality of sidewalk, air pollution, attractive sequences, environmental aesthetic, Clean environment)  Convenience: (acses to public space, Distribution of land use, physical Permeability, Visibility of signs, urban furniture)  Safety: (tarrfic, pedestrian green light, crime, slope, stairs, lighting at night) |
| 25 | JG Friesinger, A Topor, TD Bøe and IB Larsen [55] | - | Review | - | - | Social housing quality  Well-being  Social identity  Privacy |
| 26 | S Abraham Cottagiri, PJ Villeneuve, P Raina, LE Griffith, D Rainham, R Dales, CE Peters, NA Ross and DL Crouse [58] | Canada | Quantitative | 26811 | CES-D-10  SWLS | Age  Gender  Household income  Residential surrounding greenness  Frequency of neighbourhood  interactions |
| 27 | R Wang, Y Liu, Y Lu, J Zhang, P Liu, Y Yao and G Grekousis [8] | China | Quantitative | 1231 | GDS  GAI | gender  age  race  marital status  Functional ability  percieved safety  Perceived depression  and beauty  self-rated health condition  (SRH) and chronic diseases  Population density  Landuse mix  Intersection density |
| 28 | A Toma, M Hamer and A Shankar [11] | England | Quantitative | 6134 | SWLS  CES-D | Age  gender  Marital status  Occupation  Race  The length of residence  well-being  Socio-economic status |
| 29 | Y Liu, S Lu, Y Guo, HC Ho, Y Song, W Cheng, CHK Chui, OF Chan, RLH Chiu, C Webster, et al. [30] | Hong Kong | Quantitative | 2081 | GDS-15 | Age  IADL Functional ability  chronic diseases  Cognitive function  Slope of terrain  Land use mix  Social participation  Median monthly family income |
| 30 | R Wang, Y Lu, J Zhang, P Liu, Y Yao and Y Liu [29] | China | Quantitative | 1231 | GDS  GAI | Education  Gender  Age  Race  Marital status  Functional ability  Physical health conditions  Street view proportion of sky  Street view proportion of greenness  Quantity of social ties  Quality of social ties |
| 31 | J Domènech-Abella, L Switsers, J Mundó, E Dierckx, S Dury and L De Donder [26] | Belgium | Quantitative | 869 | CFAI-plus  De Jong Gierveld  scale | Being female  Marital statue  Education  Feeling lonely  Physical functioning  Mobility  Neighborhood safety  Social participation  Social cohision |
| 32 | A Curl and P Mason [28] | U.K. | Quantitative | 1071 | WEMWBS | Vehicle ownership  Age  Living alone  Cardio-respiratory health  Social contact  Neighbourhood environment quality  Neighbourhood problems(Incivilities)  Neighbourhood problems  (environmental)  Quality of local services |
| 33 | YY Chen, GHY Wong, TY Lum, VWQ Lou, AHY Ho, H Luo and TLW Tong [46] | Hong Kong | Quantitative | 400 | GDS-15 | Medical facilities  Access to Community Facility  Social support  mutual support  Instrumental support  Income |
| 34 | R Wang, Y Liu, D Xue and M Helbich [27] | China | Quantitative | 20533 | CES-D | Gender  Age  being Employed  Educational level  Married – not living with spouse, Married – living with spouse  Alcohol use  Physical health status  Green space  Population density  Sense of security  Social capital  Household income |
| 35 | S Lu, Y Liu, Y Guo, HC Ho, Y Song, W Cheng, C Chui, OF Chan, C Webster, RLH Chiu, et al. [48] | Hong Kong | Quantitative | 2081 | GDS-15  MOCA | Functional ability  Physical activity  Urban greenness  Commercial facilities Transportation,  Community facility  social activity |
| 36 | S Cho [40] | USA | Quantitative | 3684 |  | Race/Ethnicity  Gender  Age  Education  Marital status  Depression history  Self-rated health  Percieved neighborhood disorder?  Social disorder.  Social support (positive interactions with a spouse/partner, children,  relatives (i.e., other immediate family members and friend)) |
| 37 | TH Tan [41] | Malaysia | Quantitative | 510 | CES-D | GENDER  Education  ability to drive  number of dependents in the family  Home ownership  Access to facility  Natural landscape  Building attractiveness  Noise pollution  Crime related safety |
| 38 | A Barnett, CJ Zhang, JM Johnston and E Cerin [53] | - | Review | - | - | Air pollution  Density  Personal/Crime-related safety  Socio-economic status |
| 39 | M Ruiz, S Scholes and M Bobak [25] | U.K. | Quantitative | 11037 | CES-D | Age  Perceived social cohesion |
| 40 | Y Guo, SS Chang, CH Chan, Q Chang, CY Hsu and PSF Yip [24] | Hong Kong | Quantitative | 29099 | GDS-15 | Age  Gender  Poverty  Marital status  Education  Ethnic minority  Smoking status  Physical activity  Chronic illnesses,  Activity of daily living limitations  Neighborhood recreational services  walkability  Housing types  house ownership  The ratio of the elderly population in the neighborhood |
| 41 | SL Ivey, M Kealey, E Kurtovich, RH Hunter, TR Prohaska, CM Bayles and WA Satariano [44] | USA | Quantitative | 884 | CES-D | never married, divorced, separated, and widowed  self-rated health  neighborhood crime  traffic safety  social capital  inadequate financial resources |
| 42 | S Mao, N Lu and C Xiao [57] | China | Quantitative |  | CES-D | community health care  Cognitive social capital  Structural social capita |
| 43 | TG Van Tilburg, S Steinmetz, E Stolte, H van der Roest and DH de Vries [59] | Netherlands | Quantitative | 1679 | mental health inventory, self-rated health | Self-rated health/ Health improved  Urbanity of hometown  Contact frequency with children  Personally affected by own or other’s illness, deaths  Support needed, but not received  Worried about the pandemic  Trust in societal institutions increased |
| 44 | Y Li, S Su, B Luo, J Wang and S Liao [60] | China | Quantitative | 511 | PASE  PHQ-9 | Decline in Activity engagement  Worsened severity of depression |
| 45 | O Paccagnella and B Pongiglione [16] | Europe | Quantitative | 37475 | EURO-D | Age  Women  losing one’s job  Physical health status Household testing negative  death of anyone in the household being hospitalised  pre-existing depression  the risk of reporting higher levels of sadness or depression  not having or having rare personal  contacts with family and friends  lack of electronic contacts  presence of household financial fragility |
| 46 | RW-S Sit, HHK Lai, D Dong, B Wang, MC-s Wong, RY-N Chung and SY-S Wong [18] | Hong Kong | Qualitative | 27 | - | Feelings of being fearful, hopelessness, powerlessness, annoyed, frustrated, and worrisome were frequently  Worries About the Others  disrupted all types of social support  physical disconnection |
| 47 | OO Khalaf, SA Abdalgeleel and N Mostafa [17] | Arabian countries | Quantitative | 161 | FCV‑19S HADS | Age  Gender  Education  worried about having the disease  fear of COVID-19 |
